# Trends in Mortality From Drug Poisonings, Suicide, and Alcohol-Induced Deaths in the United States From 2000 to 2017

**DOI:** 10.1001/jamanetworkopen.2020.16217

**Published:** 2020-09-11

**Authors:** Meredith S. Shiels, Zaria Tatalovich, Yingxi Chen, Emily A. Haozous, Patricia Hartge, Anna M. Nápoles, Eliseo J. Pérez-Stable, Erik J. Rodriquez, Susan Spillane, David A. Thomas, Diana R. Withrow, Amy Berrington de González, Neal D. Freedman

**Affiliations:** 1 Division of Cancer Epidemiology and Genetics, National Cancer Institute, Bethesda, Maryland; 2 Division of Cancer Control and Population Sciences, National Cancer Institute, Bethesda, Maryland; 3 Pacific Institute for Research and Evaluation, Albuquerque, New Mexico; 4 Office of the Director, National Institute on Minority Health and Health Disparities, Bethesda, Maryland; 5 Division of Intramural Research, National Heart, Lung, and Blood Institute, Bethesda, Maryland; 6 Office of Research on Women’s Health, National Institutes of Health, Bethesda, Maryland

## Abstract

**Importance:**

Life expectancy has decreased in the US, driven largely by increases in drug poisoning, suicide, and alcohol-induced deaths. Assessing whether patterns of these causes differ is required to inform public health interventions.

**Objective:**

To compare patterns and trends in drug poisoning, suicide, and alcohol-induced death rates by geography and demographic characteristics.

**Design, Setting, and Participants:**

This serial cross-sectional study used national vital statistics data from the entire US population from January 1, 2000, to December 31, 2017, among US residents aged 20 to 64 years. Data were analyzed from January through August 2019.

**Exposures:**

Age, sex, race/ethnicity, county-level percentage of unemployment, rurality, and geography.

**Main Outcomes and Measures:**

Deaths were categorized as due to drug poisoning, suicide, or alcohol-induced causes based on underlying cause of death. Age-standardized incidence rates and annual percentage changes (APCs) in rates were estimated. Clusters of high-rate counties were identified with hot spot analysis. Excess deaths during 2001 to 2017 were estimated for each cause as the difference between the number of deaths observed and expected if rates had remained stable starting in 2000.

**Results:**

During 2000 to 2017, 1 446 177 drug poisoning, suicide, and alcohol-induced premature deaths occurred in the US, including 563 765 drug poisoning deaths (age-standardized rate: 17.6 per 100 000 person-years [PYs]), 517 679 suicides (age-standardized rate: 15.8 per 100 000 PYs), and 364 733 alcohol-induced deaths (age-standardized rate: 10.5 per 100 000 PYs), totaling 451 596 more deaths than expected based on 2000 rates. High drug poisoning death rates were clustered in the Northeast through Appalachia, yet rates of suicide and alcohol-induced deaths were highest in the West. Only suicide death rates were highest in rural areas. Drug poisoning death rates were highest among people aged 35 to 49 years (age-standardized rate: 23.7 per 100 000 PYs), whereas suicide and alcohol-induced death rates peaked among people aged 50 to 64 years (suicide age-standardized rate: 19.6 per 100 000 PYs; alcohol-induced age-standardized death rate: 26.8 per 100 000 PYs). Increases occurred over time across racial/ethnic groups, although trajectories and inflection years varied. Drug poisoning (2013-2017 APC, 15.0% [95% CI, 11-8%-18.3%] per year) and alcohol-induced death rates (2012-2017 APC, 4.1% [95% CI, 3.3%-4.9%] per year) have accelerated recently, while increases in suicide death rates have largely increased at a constant trajectory (2000-2017 APC, 1.8% [95% CI, 1.7%-1.9%] per year).

**Conclusions and Relevance:**

This cross-sectional study found that demographic characteristics and geographic patterns varied by cause of death, suggesting that increasing death rates from these causes were not concentrated in 1 group or region. Specialized interventions tailored for the underlying drivers of each cause of death are urgently needed.

## Key Points

**Question:**

What are the patterns and trends in US drug poisoning, suicide, and alcohol-induced premature death rates by geography and demographic characteristics?

**Findings:**

In this serial cross-sectional study using US mortality data from 2000 to 2017, including 1 446 177 drug poisoning, suicide, and alcohol-induced premature deaths, high drug poisoning death rates were clustered in the Northeast through Appalachia but high rates for suicide and alcohol-induced deaths were largely in the West. Death rates varied across these 3 causes by demographic characteristics, county-level characteristics, and over time.

**Meaning:**

This cross-sectional study found that demographic and geographic patterns varied by cause of death, suggesting that these causes of death were not concentrated in 1 group or region and tailored interventions to each cause are urgently needed.

## Teaser

This cross-sectional study compared patterns and trends in drug poisoning, suicide, and alcohol-induced premature death rates by geography and demographic characteristics.

## Introduction

Death rates in the United States have been increasing in some groups of middle-aged White and American Indian and Alaska Native men and women,^1,2,3^ largely driven by rapidly increasing death rates from drug poisonings, suicide, and alcohol-induced causes.^1,2,3,4,5^ Although all-cause midlife mortality among Black, Latino, and Asian and Pacific Islander individuals started decreasing in 1999, these decreases ended in 2009 to 2011.^4^ Increases in midlife mortality resulted in annual declines in US life expectancy from 2015 to 2017.^6,7,8^

By 2017, death rates due to drug poisonings had increased 3.6-fold and those due to suicide had increased 1.3-fold since 1999, while alcohol-induced deaths increased 1.4-fold during 1999 to 2017.^9,10,11^ Collectively, these 3 causes of death have been termed *deaths of despair* and have been largely discussed in relation to rising death rates among White men and women without a college degree.^3^ It is important to note that increases in death rates due to these causes have not been restricted to middle-aged White individuals in nonurban America. In fact, death rates from these causes increased from 2015 to 2016 across all racial/ethnic groups.^4,9,10,12^

It has been hypothesized that the root cause of these increases is societal, driven by increasing unemployment and financial insecurity.^3^ However, the underlying drivers of these causes of death are likely multifaceted and also include more distal factors (eg, access to drugs or handguns). To target interventions to high-risk groups, whether economically, clinically, or public health–focused, it is critical to understand if the highest rates and greatest increases over time in drug poisoning, suicide, and alcohol-induced death rates have occurred in the same demographic groups and geographic areas. In this study, we compared death rates and trends in rates from these 3 causes by geography, age, race, and ethnicity. We used US death certificate data for premature death (ie, ages 20-64 years) from drug poisonings, suicide, and alcohol-induced causes and conducted hot spot and trend analyses for each cause.

## Methods

This cross-sectional study used publicly available data; therefore, institutional review board approval and informed consent were not needed, per National Cancer Institute policy.

### Data Sources

Cause-specific US mortality and demographic data for January 1, 2000, to December 31, 2017, were collected from death certificates by the National Center for Health Statistics, Centers for Disease Control and Prevention. Cause of death was classified based on *International Statistical Classification of Diseases and Related Health Problems, Tenth Revision (ICD-10)* codes (eTable 1 in the Supplement).^13^ Race/ethnicity was also ascertained from death certificates and classified as non-Hispanic White (ie, White), Hispanic or Latino (ie, Latino), non-Hispanic Black (ie, Black), Asian and Pacific Islander (ie, Asian; notably <4% were Native Hawaiian or other Pacific Islander) and American Indian and Alaska Native. Population data were drawn from the US Census intercensal populations. Analyses for American Indian and Alaska Native individuals were limited to counties within the purchased/referred care delivery areas to increase accuracy of American Indian and Alaska Native race/ethnicity on death certificates.^14^ All analyses were restricted to individuals aged 20 to 64 years to focus on the age range most strongly impacted by these causes of death.

County-level percentages of people who were unemployed (ie, percentage of civilians aged ≥16 years in the labor force who were unemployed) were ascertained from the 2013 to 2017 Census American Community Survey and classified in quintiles based on population distribution across counties. Counties were categorized using a collapsed version of the 2013 Rural-Urban Continuum codes developed by the Department of Agriculture.^15^

### Statistical Analysis

We compared age-standardized death rates from 2013 to 2017 and annual percentage changes (APCs) in age-standardized death rates from 2000 to 2017 to assess whether the highest absolute rates and most rapid increases in death rates were the same or different for drug poisonings, suicide, and alcohol-induced deaths across demographic groups and county-level characteristics. Thus, age-standardized drug poisoning, suicide, and alcohol-induced death rates were estimated by sex, age group (ie, 20-34, 35-49, and 50-64 years), race/ethnicity, county-level percentage of unemployment (ie, ≤5.10%, 5.11%-5.95%, 5.96%-6.89%, 6.90%-7.96%, and ≥7.97%) and county rural/urban status (ie, metropolitan with ≥1 million people, metropolitan with 250 000 to <1 million people, metropolitan with <250 000 people, urban with ≥20 000 people, urban with 2500 to <20 000 people, and completely rural with <2500 people). All rates were standardized in 5-year age groups to the 2000 US population, as is standard practice in mortality trends analyses.^11^ The number of excess deaths occurring during 2001 to 2017 was estimated as the difference between the number of observed deaths and the number of deaths expected if rates had remained stable from 2000 to 2017. Expected deaths were estimated by multiplying age-specific 2000 death rates by the population size in each subsequent year. Hot spot analysis was used to identify clusters of counties within the US with significantly higher or lower age-standardized death rates for each cause during 2013 to 2017 with the Hot Spot Analysis (Getis-Ord Gi*) method^16^ using ArcGIS Pro version 2.2 (Esri).

To compare changes over time in drug poisoning, suicide, and alcohol-induced death rates, we estimated APCs and average APCs in death rates during 2000 to 2017 using Joinpoint regression software.^17^ Joinpoint uses a log-linear model to estimate APCs for segments of calendar years defined based on statistically significant changes in rate trajectories over time. Pairwise comparisons of APCs for each cause of death were assessed with a test for parallelism. *P* values were 2-sided, and statistical significance was set at *P* < .05. Data were analyzed from January through August 2019.

## Results

### Differences by Population Characteristics

During 2000 to 2017, 563 765 drug poisoning deaths (age-standardized rate: 17.6 deaths per 100 000 person-years [PYs]), 517 679 suicides (age-standardized rate: 15.8 deaths per 100 000 PYs) and 364 733 alcohol-induced deaths (age-standardized rate: 10.5 deaths per 100 000 PYs) occurred among individuals aged 20 to 64 years (1.8 billion PYs of follow-up) in the United States.

**Figure 1.**
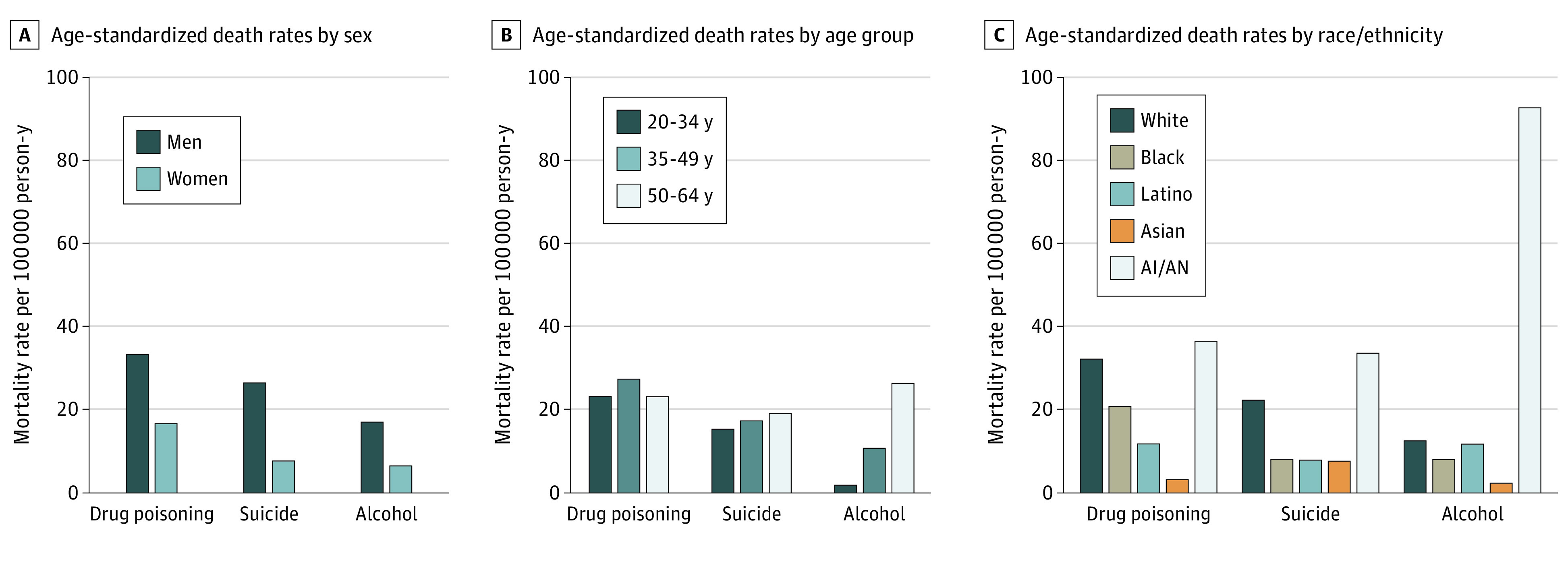
Age-Standardized Death Rates Per 100 000 Person-Years for Drug Poisoning, Suicide, and Alcohol-Induced Deaths From 2013 to 2017 AI/AN indicates American Indian and Alaska Native.

In 2013 to 2017, death rates were consistently higher among men than women for each cause-of-death (Figure 1A). Drug poisoning death rates were highest among individuals aged 35 to 49 years (age-standardized rate: 23.7 deaths per 100 000 PYs), whereas suicide and alcohol-induced death rates increased with age. Suicide death rates peaked among people aged 50 to 64 years (age-standardized rate: 19.6 deaths per 100 000 PYs). Alcohol-induced death rates among individuals aged 50 to 64 years (age-standardized rate: 26.8 deaths per 100 000 PYs) were on par with drug poisoning death rates among those aged 35 to 49 years (Figure 1B).

In 2013 to 2017, drug poisoning death rates were highest among American Indian and Alaska Native individuals (36.9 deaths per 100 000 PYs) and White individuals (32.6 deaths per 100 000 PYs), followed by Black individuals (21.1 deaths per 100 000 PYs). Suicide rates were highest among American Indian and Alaska Native individuals (34.0 deaths per 100 000 PYs) and White individuals (22.7 deaths per 100 000 PYs) and far exceed those in the other racial/ethnic groups (approximately 8 deaths per 100 000 PYs). Alcohol-induced death rates were higher among American Indian and Alaska Native individuals (93.1 deaths per 100 000 PYs) than White individuals (12.9 deaths per 100 000 PYs) and Latino individuals (12.2 deaths per 100 000 PYs), the 2 groups with the next highest rates (Figure 1C).

### Differences by Context

**Figure 2.**
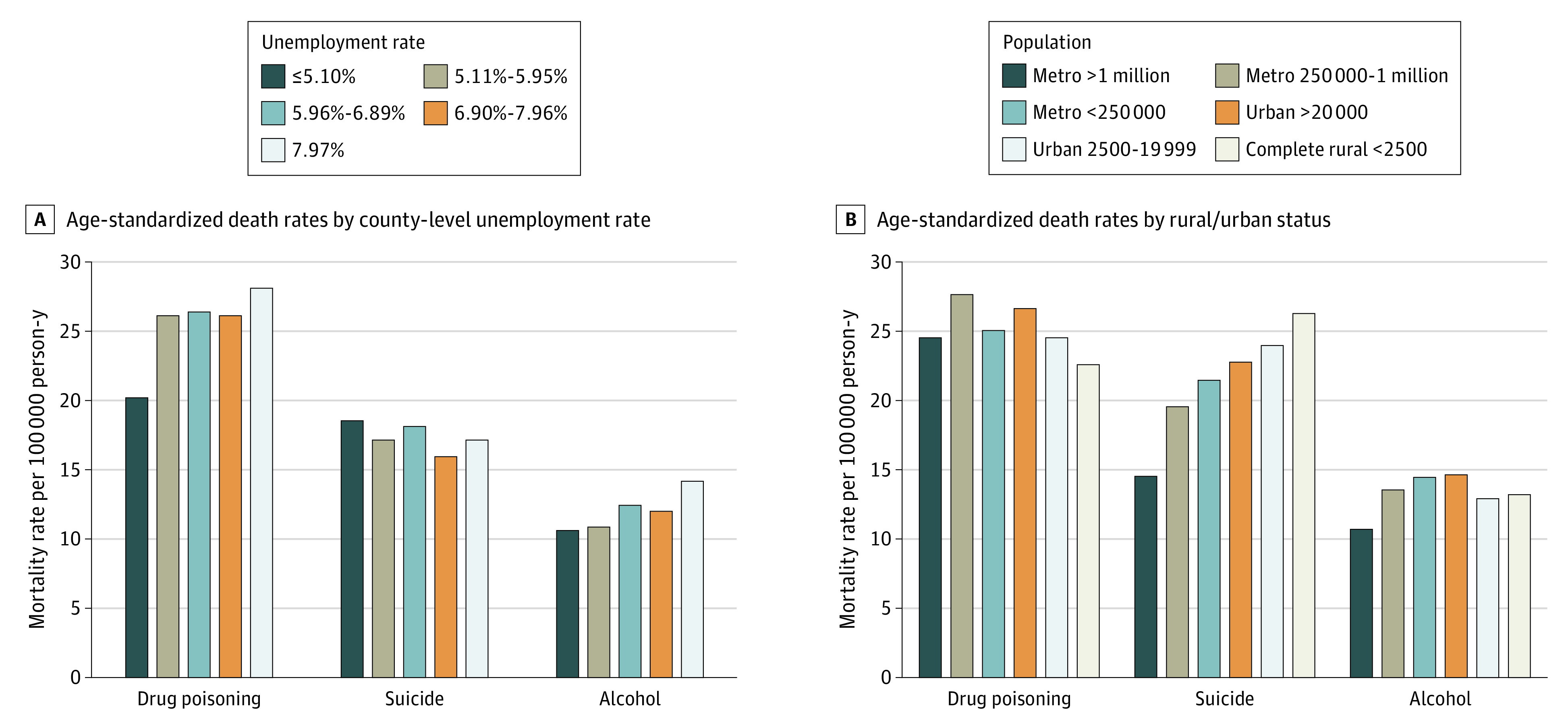
Age-Standardized Death Rates Per 100 000 Person-Years for Drug Poisoning, Suicide, and Alcohol-Induced Deaths From 2013 to 2017

Stratified by county-level percentage of unemployment, drug poisoning and alcohol-induced death rates were highest in counties in the highest quintile of unemployment and lowest in the lowest quintile. However, suicide rates were highest in counties in the lowest quintile of unemployment and lowest in the second highest quintile of unemployment (Figure 2A).

Patterns differed between urban and rural counties (Figure 2B). Drug poisoning death rates were similar with no clear gradient by rurality (range, 22.7-27.8 deaths per 100 000 PYs), whereas suicide rates were highest in rural counties (age-standardized death rate: 26.4 per 100 000 PYs), and alcohol-induced deaths were highest in the smallest metropolitan (age-standardized death rate: 14.6 per 100 000 PYs) and largest urban (age-standardized death rate: 14.6 per 100 000 PYs) counties. Suicide rates were higher than drug poisoning death rates in rural counties.

**Figure 3.**
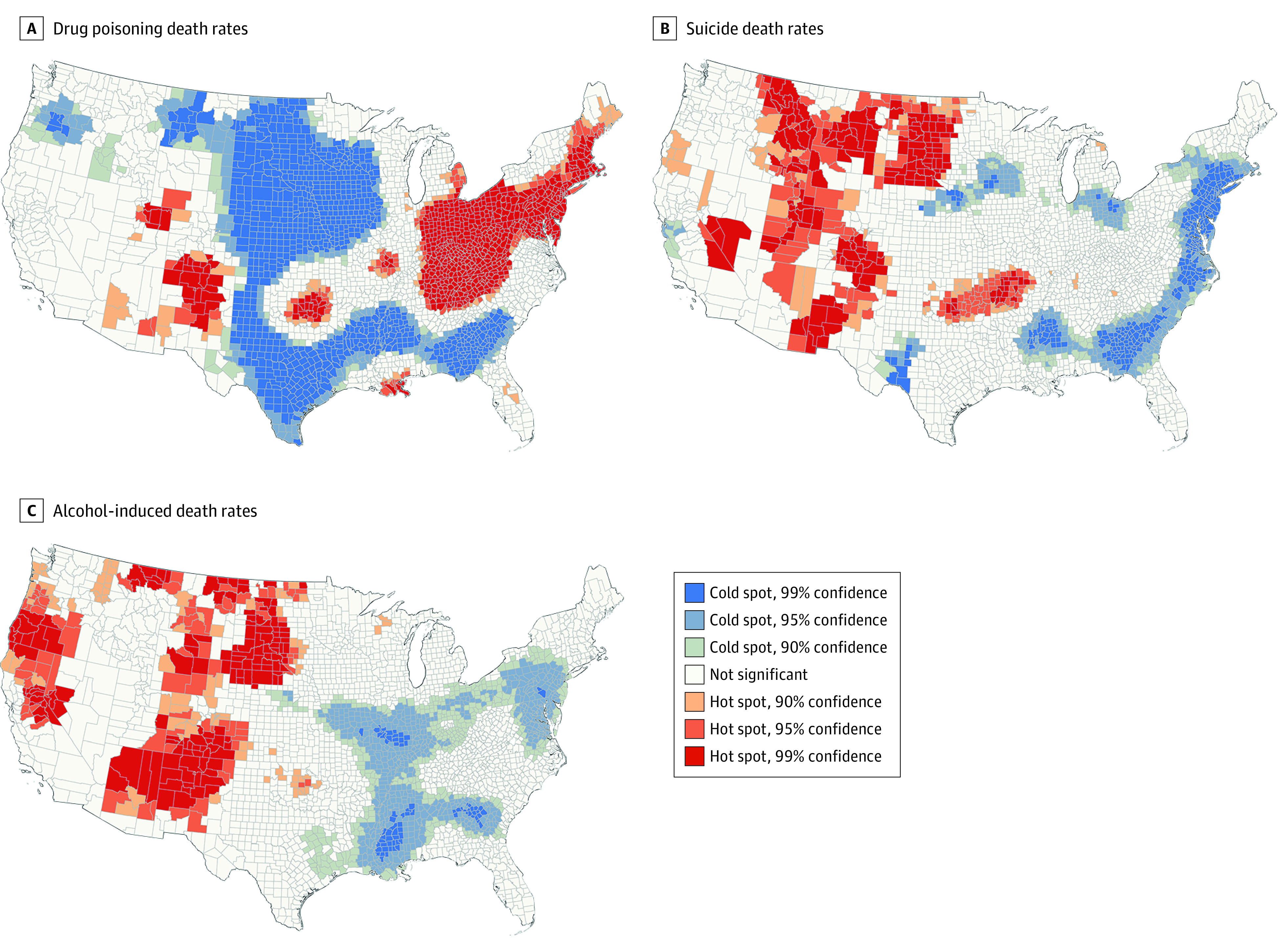
Hot Spot Analysis From 2013 to 2017

Hot spot analysis identified statistically significant clusters of US counties with high (hot spots) and low (cold spots) drug poisoning, suicide, and alcohol-induced death rates (Figure 3). The largest significant cluster of counties with elevated drug poisoning death rates extended from the Northeast into Ohio, Indiana, Kentucky, Tennessee, West Virginia, and parts of Virginia and North Carolina. Additional significant hot spots were identified in New Mexico, Colorado, Utah, and Oklahoma. In contrast, the significant hot spots in suicide and alcohol-induced death rates were largely confined to the western half of the US, with hot spots for both causes of death from Montana and North Dakota to New Mexico and Arizona. Hot spots for of all 3 causes were present in New Mexico and Colorado.

For drug poisoning deaths, there were significant clusters of counties with lower death rates that extended from North Dakota and Minnesota south through Texas and then east from Texas to Georgia and South Carolina. Lower suicide rates clustered along the East coast, and lower alcohol-induced death rates clustered from Missouri east through Pennsylvania and from Louisiana east through Georgia. Cold spots for all three causes were present in Mississippi, Alabama, and Georgia.

### Trends Over Time

Statistically significant differences in APCs were observed across causes of death (eTable 2 in the Supplement). Drug poisoning death rates increased 11.4% (95% CI, 8.7%-14.2%) per year during 2000 to 2006, 2.5% (95% CI, 0.6%-4.5%) per year during 2006 to 2013, and sharply accelerated to 15.0% (95% CI, 11.8%-18.3%) per year during 2013 to 2017 (eFigure in the Supplement; Table). In contrast, alcohol-induced death rates started increasing during 2005 to 2012 (APC, 2.1% [95% CI, 1.5%-2.8%] per year) and accelerated to 4.1% (95% CI, 3.3%-4.9%) per year during 2012 to 2017. Suicide rates increased steadily at 1.8% (95% CI, 1.7%-1.9%) per. These increases indicate an additional 451 596 deaths, including 317 848 drug poisoning deaths, 85 769 suicide deaths, and 47 978 alcohol-induced deaths, during 2001 to 2017 more than what would have occurred if the death rates in 2000 had persisted.

**Table.** Annual Percentage Changes in Drug Poisoning, Suicide, and Alcohol-Induced Death Rates by Sex, Age Group, and Race/Ethnicity From 2000 to 2017

Cause of death	Average APC from 2000-2017)	Segment 1^a^		Segment 2^a^		Segment 3^a^		Segment 4^a^	
		Years	APC (95% CI)	Years	APC (95% CI)	Years	APC (95% CI)	Years	APC (95% CI)
**Overall**									
Drug poisoning	8.5 (7.2 to 9.8)	2000-2006	11.4 (8.7 to 14.2)	2006-2013	2.5 (0.6 to 4.5)	2013-2017	15.0 (11.8 to 18.3)	NA	NA
Suicide	1.8 (1.7 to 1.9)	2000-2017	1.8 (1.7 to 1.9)	NA	NA	NA	NA	NA	NA
Alcohol	2.0 (1.7 to 2.4)	2000-2005	−0.1 (−1.0 to 0.8)	2005-2012	2.1 (1.5 to 2.8)	2012-2017	4.1 (3.3 to 4.9)	NA	NA
**Men**									
Drug poisoning	8.3 (7.0 to 9.7)	2000-2006	10.0 (7.3 to 12.8)	2006-2013	1.9 (0.0 to 4.0)	2013-2017	17.7 (14.3 to 21.2)	NA	NA
Suicide	1.6 (1.4 to 1.7)	2000-2017	1.6 (1.4 to 1.7)	NA	NA	NA	NA	NA	NA
Alcohol	1.5 (1.1 to 1.9)	2000-2005	−0.3 (−1.2 to 0.6)	2005-2011	1.3 (0.5 to 2.2)	2011-2017	3.2 (2.6 to 3.8)	NA	NA
**Women**									
Drug poisoning	9.7 (8.1 to 11.3)	2000-2002	27.0 (12.6 to 43.2)	2002-2007	10.2 (7.2 to 13.2)	2007-2014	3.9 (2.6 to 5.1)	2014-2017	12.0 (8.7 to 15.4)
Suicide	2.6 (2.5 to 2.8)	2000-2017	2.6 (2.5 to 2.8)	NA	NA	NA	NA	NA	NA
Alcohol	3.4 (2.9 to 4.0)	2000-2007	1.4 (0.3 to 2.6)	2007-2017	4.8 (4.3 to 5.4)	NA	NA	NA	NA
**Age 20-34 y**									
Drug poisoning	10.2 (8.6 to 11.9)	2000-2006	13.8 (10.2 to 17.6)	2006-2013	3.3 (1.0 to 5.8)	2013-2017	17.6 (13.7 to 21.5)	NA	NA
Suicide	2.0 (1.4 to 2.6)	2000-2008	0.7 (0.1 to 1.3)	2008-2014	2.2 (1.1 to 3.4)	2014-2017	5.1 (2.7 to 7.5)	NA	NA
Alcohol	4.8 (3.3 to 6.3)	2000-2005	0.1 (−4.6 to 5.1)	2005-2017	6.8 (5.8 to 7.9)	NA	NA	NA	NA
**Age 35-49 y**									
Drug poisoning	7.0 (5.4 to 8.6)	2000-2003	14.2 (6.0 to 23.0)	2003-2014	2.4 (1.4 to 3.4)	2014-2017	17.8 (11.9 to 23.9)	NA	NA
Suicide	1.2 (1.1 to 1.4)	2000-2017	1.2 (1.1 to 1.4)	NA	NA	NA	NA	NA	NA
Alcohol	0.6 (0.2 to 1.1)	2000-2005	−1.7 (−2.7 to −0.7)	2005-2013	0.5 (−0.1 to 1.2)	2013-2017	3.8 (2.4 to 5.3)	NA	NA
**Age 50-64 y**									
Drug poisoning	11.6 (10.3 to 12.9)	2000-2006	18.5 (15.2 to 22.0)	2006-2014	6.0 (4.6 to 7.4)	2014-2017	13.5 (9.3 to 17.9)	NA	NA
Suicide	2.3 (1.3 to 3.4)	2000-2006	2.7 (1.5 to 3.9)	2006-2009	5.1 (−0.6 to 11.2)	2009-2015	1.7 (0.6 to 2.9)	2015-2017	−1.0 (−5.7 to 3.9)
Alcohol	2.9 (2.5 to 3.2)	2000-2010	2.0 (1.8 to 2.3)	2010-2015	4.8 (4.0 to 5.6)	2015-2017	2.4 (0.2 to 4.6)	NA	NA
**White**									
Drug poisoning	9.9 (8.5 to 11.3)	2000-2006	13.7 (10.7 to 16.9)	2006-2014	4.5 (2.9 to 6.2)	2014-2017	17.2 (11.7 to 22.9)	NA	NA
Suicide	2.5 (1.5 to 3.5)	2000-2002	4.5 (−0.2 to 9.3)	2002-2005	0.7 (−3.6 to 5.2)	2005-2008	4.0 (−0.3 to 8.5)	2008-2017	2.2 (1.8 to 2.6)
Alcohol	3.1 (2.8 to 3.4)	2000-2006	1.7 (1.1 to 2.3)	2006-2013	3.2 (2.7 to 3.8)	2013-2017	5.0 (4.0 to 60)	NA	NA
**Black**									
Drug poisoning	6.6 (3.8 to 9.5)	2000-2006	6.3 (2.9 to 9.7)	2006-2010	−7.1 (−14.8 to 1.3)	2010-2014	7.2 (−1.4 to 16.6)	2014-2017	27.9 (20.2 to 36.2)
Suicide	1.3 (0.7 to 1.9)	2000-2008	−0.6 (−1.0 to −0.3)	2008-2012	2.4 (0.8 to 4.0)	2012-2015	−0.2 (−3.2 to 2.8)	2015-2017	9.6 (6.6 to 12.6)
Alcohol	−2.0 (−2.7 to −1.3)	2000-2006	−6.8 (−7.8 to −5.7)	2006-2012	−1.0 (−2.6 to 0.6)	2012-2017	2.9 (1.3 to 4.5)	NA	NA
**Latino**									
Drug poisoning	5.1 (3.7 to 6.5)	2000-2014	2.2 (1.2 to 3.2)	2014-2017	19.6 (11.5 to 28.4)	NA	NA	NA	NA
Suicide	1.5 (0.9 to 2.1)	2000-2013	0.5 (0.0 to 1.0)	2013-2017	4.8 (2.5 to 7.3)	NA	NA	NA	NA
Alcohol	−0.1 (−0.7 to 0.6)	2000-2004	−3.2 (−5.3 to −1.1)	2004-2012	−0.1 (−0.9 to 0.7)	2012-2017	2.6 (1.4 to 3.7)	NA	NA
**Asian**									
Drug poisoning	10.1 (7.8 to 12.3)	2000-2005	13.2 (8.5 to 18.2)	2005-2009	2.8 (−4.2 to 10.3)	2009-2014	8.3 (4.4 to 12.3)	2014-2017	18.2 (13.3 to 23.2)
Suicide	1.9 (1.5 to 2.3)	2000-2017	1.9 (1.5 to 2.3)	NA	NA	NA	NA	NA	NA
Alcohol	2.5 (1.7 to 3.2)	2000-2017	2.5 (1.7 to 3.2)	NA	NA	NA	NA	NA	NA
**American Indian and Alaska Native**									
Drug poisoning	10.1 (7.4 to 12.8)	2000-2004	25.5 (12.5 to 40.0)	2004-2017	5.7 (4.6 to 6.9)	NA	NA	NA	NA
Suicide	3.1 (2.5 to 3.6)	2000-2017	3.1 (2.5 to 3.6)	NA	NA	NA	NA	NA	NA
Alcohol	4.0 (3.6 to 4.5)	2000-2017	4.0 (3.6 to 4.5)	NA	NA	NA	NA	NA	NA

Abbreviations: APC, annual percentage change; NA, not applicable.

^a^ Segments were chosen by Joinpoint regression.

Of the 3 causes of death, drug poisoning deaths increased the most rapidly among both men (average APC, 8.3% [95% CI, 7.0%-9.7%] per year) and women (average APC, 9.7% [95% CI, 8.1%-11.3%] per year), with the largest increases observed during 2013 or 2014 to 2017 (Table). Suicide rates increased 1.6% (95% CI, 1.4%-1.7%) per year among men and 2.6% (95% CI, 2.5%-2.8%) per year among women throughout the time period. Alcohol-induced death rates began increasing in 2005 among men and in 2000 among women and accelerated in more recent years (men: 2011-2017 APC, 3.2% [95% CI, 2.6%-3.8%] per year; women: 2007-2017 APC, 4.8% [95% CI, 4.3%-5.4%] per year). For each cause of death, APCs differed significantly from each other among men and women (eTable 2 in the Supplement).

Drug poisoning death rates increased in each racial/ethnic group during 2000 to 2017, except for Black individuals, among whom rates declined during 2006 to 2010, and then increased (Table; eFigure in the Supplement). Suicide rates increased 1.9% (95% CI, 1.5%-2.3%) per year among Asian individuals and 3.1% (95% CI, 2.5%-3.6%) per year among American Indian and Alaska Native individuals during 2000 to 2017. Among Latino individuals, suicide rates increased 0.5% (95% CI, 0%-1.0%) per year during 2000 to 2013 and 4.8% (95% CI, 2.5%-7.3%) per year during 2013 to 2017. Increases in suicide rates among White and Black individuals were more variable over time, with significant increases observed in both groups in the most recent time period (White individuals from 2008-2017: 2.2% [95% CI, 1.8%-2.6%] per year; Black individuals from 2015 to 2017: 9.6% [95% CI, 6.6%-12.6%] per year). While alcohol-induced death rates significantly increased across 2000 to 2017 among White (APC, 3.1% [95% CI, 2.8%-3.4%], Asian (APC, 2.5% [95% CI, 1.7%-3.2%] per year), and American Indian and Alaska Native (APC, 4.0% [95% CI, 3.6% to 4.5%] per year) individuals, significant increases were only observed during 2012 to 2017 among Black individuals (APC, 2.9% [95% CI, 1.3%-4.5%] per year) and Latino individuals (APC, 2.6% [95% CI, 1.4%-3.7%] per year). Annual percentage changes in death rates due to drug poisonings, suicide, and alcohol-related causes differed significantly within each racial/ethnic group (eTable 2 in the Supplement), except for APCs for suicide and alcohol-induced causes among Asian individuals, which were parallel.

**Figure 4.**
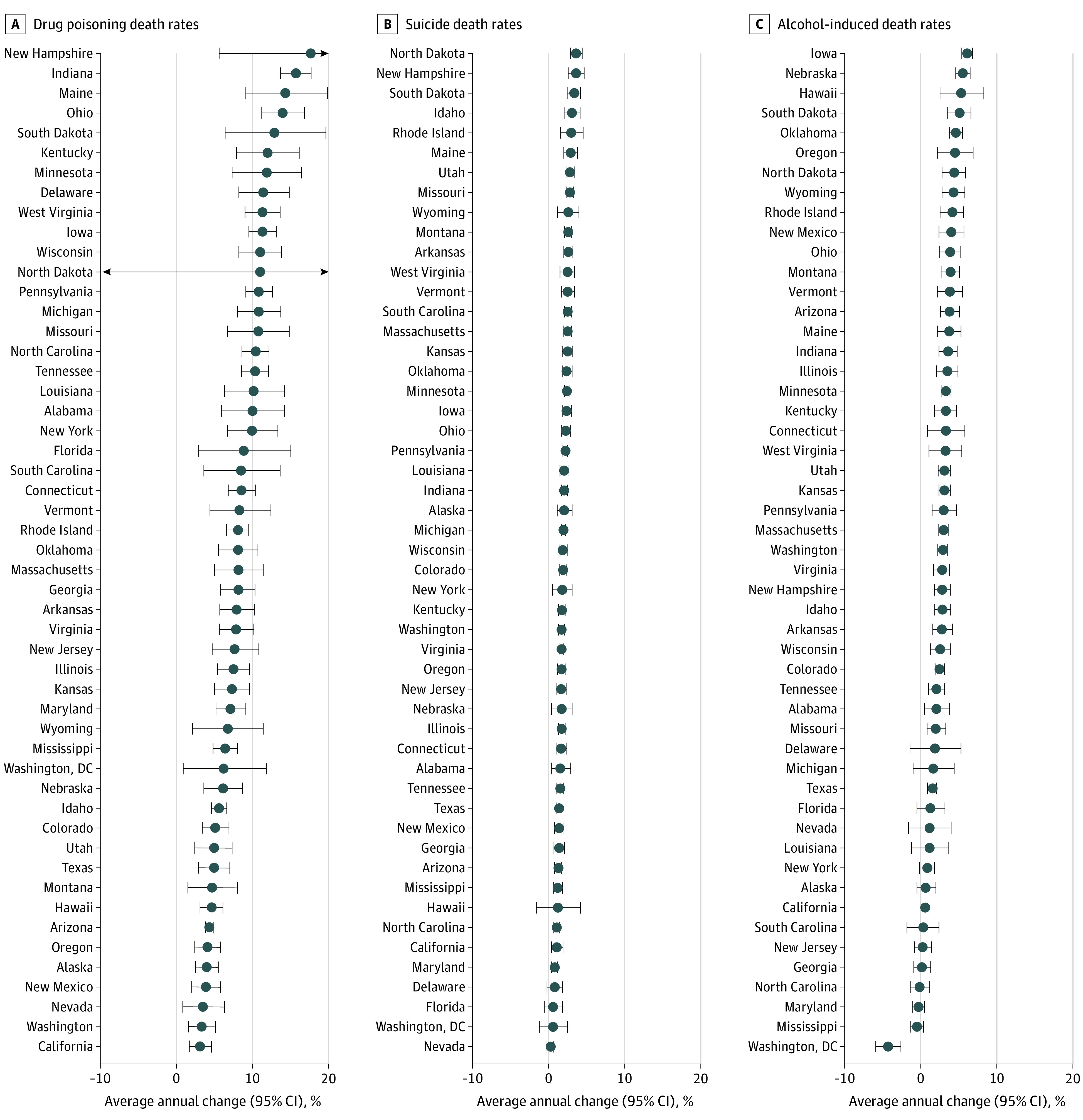
Average Annual Percentage Change by US State and Washington, District of Columbia (DC) From 2000 to 2017 Points represent average annual percentage changes; lines, 95% CIs.

Drug poisoning (average APC range, 3.1% to 17.6% per year), suicide (average APC range, 0.3% to 3.6% per year), and alcohol-induced death rates (average APC range, −4.3% to 6.1% per year) increased significantly during 2000 to 2017 in nearly every state and Washington, District of Columbia. (Figure 4). However, the states with the most rapid increases differed by cause of death. The most rapid increases were observed in New Hampshire (average APC, 17.6% [95% CI, 5.6%-30.9%] per year) and Indiana (average APC, 15.7% [95% CI, 13.7%-17.7%] per year) for drug poisoning deaths, North Dakota (average APC, 3.6% [95% CI, 2.9%-4.4%] per year) and New Hampshire (average APC, 3.6% [95% CI, 2.6%-4.7%] per year) for suicide, and Iowa (average APC, 6.1% [95% CI, 5.4%-6.8%] per year) and Nebraska (average APC, 5.5% [95% CI, 4.6%-6.5%] per year) for alcohol-induced deaths.

## Discussion

This cross-sectional study found that drug poisoning, suicide, and alcohol-induced death rates each increased dramatically among individuals aged 20 to 64 years in the US during 2000 to 2017. However, the demographic groups and geographic areas with the highest death rates and strongest increases over time differed by cause of death. Thus, these 3 causes of death should be considered separately when targeting public health interventions toward populations at the highest risk. Furthermore, it is important to reiterate that these increases are not limited to middle-aged White men and women, as they have impacted all racial/ethnic groups in recent years, nearly every US state, and rural and urban communities.

Of striking public health concern, drug poisoning death rates have increased rapidly, accelerating to 15.0% per year during 2013 to 2017. Although individual drugs were not specifically examined here, this development largely reflects the ongoing opioid epidemic, which has been classified as having 3 waves.^18^ The first began in the 1990s with deaths due to overprescription of opioids for pain management.^18^ In 2010, heroin-related death rates began to increase rapidly followed by a surge in fentanyl-related deaths starting in 2013. Cocaine and psychostimulant-related death rates have also increased.^19,20^ American Indian and Alaska Native individuals and White individuals had the highest drug poisoning death rates; however, rates have increased rapidly among all racial/ethnic groups, particularly during 2013 to 2017. Although rates were highest and increased most rapidly in the Northeast and Appalachia, drug poisoning death rates increased significantly in nearly every state and have affected rural and urban counties. Importantly, drug poisoning death rates are also increasing in several other high-income countries.^21^

Although suicide rates increased across all racial/ethnic groups, they were highest among American Indian and Alaska Native individuals, followed by White individuals. In rural counties, suicide rates were highest and exceeded drug poisoning death rates. Rural and urban differences in suicide have increased over time, with more rapid increases occurring in rural counties.^10^ Half of all suicides in the US are carried out with firearms.^22^ Firearm-related suicides were more frequent and increased more rapidly in nonmetropolitan rural counties than metropolitan counties.^23^ A 2018 study^24^ found an inverse association between stronger state firearm laws and suicides. Although suicides by drug poisoning have increased, they comprise only 13% of suicides, with only approximately one-third due to opioids.^25^

Increases in alcohol-induced death rates began more recently (in 2005) than drug poisoning deaths and suicides and accelerated during 2012 to 2017. Rates of alcohol-induced deaths were highest during 2013 to 2017 among individuals aged 50 to 64 years but increased most rapidly among individuals aged 20 to 34 years and are exceedingly high among American Indian and Alaska Native individuals. Increasing alcohol-induced death rates are consistent with the increasing prevalence of alcohol use, high-risk drinking, and alcohol-use disorders.^26,27^ As most alcohol-induced deaths were coded as alcoholic liver disease or other conditions driven by chronic alcohol use and progressive liver damage, it is likely that the more recent trends in death rates reflect a mixture of short- and long-term effects of excessive alcohol consumption.

It has been proposed that worsening opportunities in the labor market among White individuals, particularly those with no more than a high school education, have contributed to increasing death rates from drug poisoning, suicide, and alcohol during middle age.^3^ However, other factors likely also contribute.^28,29,30^ Increasing drug poisoning, suicide, and alcohol-induced death rates in the US are not limited to White individuals; furthermore, demographic and geographic patterns of these 3 causes of death differ substantially, indicating a more nuanced and complex picture. Our findings indicate that these 3 causes of death merit individual consideration, and their underlying causes and optimal prevention strategies may differ in nature, intensity, and duration across populations and contexts.

While it is likely that increasing economic opportunities and wages and reducing inequalities would result in health improvements, prior work has shown that economic policies have not had a universal impact on rates of drug poisoning, suicide, and alcohol-induced deaths. For example, higher minimum wages and the earned income tax credit have been shown to reduce suicides but not drug poisoning deaths among adults with lower educational attainment.^31,32^ Another study by Ruhm^33^ found that economic factors are likely to explain only a small fraction of drug poisoning deaths, concluding that the availability and use of drugs is more likely to be the key driver. Ruhm^33^ found no association of economic factors with suicide and alcohol-induced death rates. Drug and alcohol poisoning and chronic liver disease death rates increased most rapidly in the most economically insecure counties, while suicide rates increased uniformly regardless of county-level economic insecurity.^34^

Many deaths due to drug poisoning, suicide, and alcohol-induced causes may be broadly associated with underlying feelings of despair, whether driven by lack of economic opportunity or other factors. However, each cause of death also reflects access to drugs, alcohol, firearms, and other means of suicide. Although some policies aimed at prevention can be applied universally, population- and epidemic-specific targeted interventions are likely also needed. For drug poisonings, guidelines for safer opioid prescribing and use of medication-assisted treatment for substance use disorder, expansion of health care insurance coverage to include substance use disorder treatment, criminal justice reform, and expanded access to naloxone are policies that could help address the epidemic.^35,36^ Efforts focused on stopping drug trafficking are also critical. In 2017, the Centers for Disease Control and Prevention released recommendations for suicide prevention, including strengthening economic supports, increasing access to and delivery of preventive suicide care, creating protective environments, promoting connectedness, teaching coping and problem-solving skills, and identifying and supporting people at risk.^37^ People who have previously attempted suicide and those with severe depression are high-risk populations to target for suicide prevention interventions. Policies focused on reducing firearm access during periods of high suicide risk have also been recommended.^38^ Finally, the US Preventive Services Task Force recommends that primary care clinicians screen adults for unhealthy alcohol use and provide behavioral counseling interventions to those who engage in hazardous drinking.^39^ As American Indian and Alaska Native populations have the highest rates for all 3 causes, there may be alternative explanatory factors associated with the historical trauma in these communities, and interventions to address these disparities are needed.

### Strengths and Limitations

The main strength of our analysis is the use of nationwide death certificate data to examine patterns and trajectories in drug poisoning, suicide, and alcohol-induced death rates. The comparisons presented here show a clear juxtaposition of these 3 causes of death and support an urgent need for appropriately targeted interventions. There are inherent limitations in the use of death certificate data, including potential misclassification of causes of death and race/ethnicity^40^; however, we restricted our analysis to purchased/referred care delivery areas to mitigate misclassification of American Indian and Alaska Native individuals.^14^ Although this approach is recommended to increase the sensitivity of ascertaining American Indian and Alaska Native individuals in mortality data, our results may not be generalizable to American Indian and Alaska Native individuals living outside of purchased/referred care delivery areas. Furthermore, 10% of drug poisoning deaths were classified as undetermined intent, which may include misclassified suicides, although it is unlikely that this had a major impact on the trends for either cause of death. Alcohol-induced deaths only include those deaths that are most clearly associated with alcohol use and do not consider other causes of death (eg, certain cancers, motor vehicle crashes) for which alcohol is a strong contributor, thus underestimating the true mortality burden due to alcohol.

## Conclusion

This cross-sectional study found alarming recent increases in drug poisoning, suicide, and alcohol-induced death rates that differed substantially by demographic and geographic factors in the US. Each of these causes of death represents increasing and complex causal factors requiring targeted efforts at multiple levels to reverse alarming trends, reach national and international public health goals, and catch up with the steady progress in life expectancy occurring in other high-income countries.

## Notes

Supplement. **eTable 1.** *International Statistical Classification of Diseases and Related Health Problems, Tenth Revision* (*ICD-10*) Codes for Drug Poisoning, Suicide and Alcohol-Induced Deaths **eTable 2.** *P *Values for Test of Parallelism **eFigure.** Trends in Drug Poisoning, Suicide and Alcohol-Induced Death Rates Among Individuals Aged 20 to 64 Years in the United States From 2000 to 2017 Stratified by Sex and Race/Ethnicity

## References

[1] Shiels MS, Chernyavskiy P, Anderson WF, Trends in premature mortality in the USA by sex, race, and ethnicity from 1999 to 2014: an analysis of death certificate data. Lancet. 2017;389(10073):1043-1054. doi:10.1016/S0140-6736(17)30187-3 28131493 PMC5388357

[2] Case A, Deaton A Rising morbidity and mortality in midlife among white non-Hispanic Americans in the 21st century. Proc Natl Acad Sci U S A. 2015;112(49):15078-15083. doi:10.1073/pnas.1518393112 26575631 PMC4679063

[3] Case A, Deaton A Mortality and morbidity in the 21^st^ century. Brookings Pap Econ Act. 2017;2017:397-476. doi:10.1353/eca.2017.0005 29033460 PMC5640267

[4] Woolf SH, Chapman DA, Buchanich JM, Bobby KJ, Zimmerman EB, Blackburn SM Changes in midlife death rates across racial and ethnic groups in the United States: systematic analysis of vital statistics. BMJ. 2018;362:k3096. doi:10.1136/bmj.k3096 30111554 PMC6092678

[5] Stein EM, Gennuso KP, Ugboaja DC, Remington PL The epidemic of despair among White Americans: trends in the leading causes of premature death, 1999-2015. Am J Public Health. 2017;107(10):1541-1547. doi:10.2105/AJPH.2017.303941 28817333 PMC5607670

[6] Kochanek KD, Murphy S, Xu J, Arias E Mortality in the United States, 2016. NCHS Data Brief. 2017;(293):1-8. 29319473

[7] Murphy SL, Xu J, Kochanek KD, Arias E Mortality in the United States, 2017. NCHS Data Brief. 2018;(328):1-8. 30500322

[8] Xu J, Murphy SL, Kochanek KD, Arias E Mortality in the United States, 2015. NCHS Data Brief. 2016;(267):1-8. 27930283

[9] Hedegaard H, Miniño AM, Warner M Drug overdose deaths in the United States, 1999-2017. NCHS Data Brief. 2018;(329):1-8. 30500323

[10] Hedegaard H, Curtin SC, Warner M Suicide mortality in the United States, 1999-2017. NCHS Data Brief. 2018;(330):1-8. 30500324

[11] Kochanek KD, Murphy SL, Xu J, Arias E Deaths: final data for 2017. Natl Vital Stat Rep. 2019;68(9):1-77. 32501199

[12] QuickStats Age-adjusted death rates* attributable to alcohol-induced causes,^†^ by race/ethnicity—United States, 1999-2015. MMWR Morb Mortal Wkly Rep. 2017;66(18):491. doi:10.15585/mmwr.mm6618a12 28493858 PMC5657989

[13] World Health Organization International Statistical Classification of Diseases, Tenth Revision (ICD-10). World Health Organization; 1992.

[14] Jim MA, Arias E, Seneca DS, Racial misclassification of American Indians and Alaska Natives by Indian Health Service Contract Health Service Delivery Area. Am J Public Health. 2014;104(suppl 3):S295-S302. doi:10.2105/AJPH.2014.301933 24754617 PMC4035863

[15] US Department of Agriculture Economic Research Service Rural–urban continuum codes. Accessed December 4, 2018. https://www.ers.usda.gov/data-products/rural-urban-continuumcodes.aspx

[16] Getis A, Ord JK The analysis of spatial association by use of distance statistics. Geographical Analysis. 1992;24(3):189-206. doi:10.1111/j.1538-4632.1992.tb00261.x

[17] Kim HJ, Fay MP, Feuer EJ, Midthune DN Permutation tests for joinpoint regression with applications to cancer rates. Stat Med. 2000;19(3):335-351. doi:10.1002/(SICI)1097-0258(20000215)19:3<335::AID-SIM336>3.0.CO;2-Z 10649300

[18] Centers for Disease Control and Prevention Opioid overdose: understanding the epidemic. Accessed May 6, 2019. https://www.cdc.gov/drugoverdose/epidemic/index.html

[19] Shiels MS, Freedman ND, Thomas D, Berrington de Gonzalez A Trends in U.S. drug overdose deaths in non-Hispanic Black, Hispanic, and non-Hispanic White persons, 2000-2015. Ann Intern Med. 2018;168(6):453-455. doi:10.7326/M17-1812 29204603 PMC6309971

[20] Kariisa M, Scholl L, Wilson N, Seth P, Hoots B Drug overdose deaths involving cocaine and psychostimulants with abuse potential—United States, 2003-2017. MMWR Morb Mortal Wkly Rep. 2019;68(17):388-395. doi:10.15585/mmwr.mm6817a3 31048676 PMC6541315

[21] Chen Y, Shiels MS, Thomas D, Freedman ND, Berrington de González A Premature mortality from drug overdoses: a comparative analysis of 13 Organisation for Economic Co-operation and Development member countries with high-quality death certificate data, 2001 to 2015. Ann Intern Med. 2019;170(5):352-354. doi:10.7326/M18-2415 30422274 PMC8294457

[22] Centers for Disease Control and Prevention National Center for Health Statistics Suicide and self-inflicted injury. Accessed September 23, 2019. https://www.cdc.gov/nchs/fastats/suicide.htm

[23] Ivey-Stephenson AZ, Crosby AE, Jack SPD, Haileyesus T, Kresnow-Sedacca MJ Suicide trends among and within urbanization levels by sex, race/ethnicity, age group, and mechanism of death—United States, 2001-2015. MMWR Surveill Summ. 2017;66(18):1-16. doi:10.15585/mmwr.ss6618a1 28981481 PMC5829833

[24] Kaufman EJ, Morrison CN, Branas CC, Wiebe DJ State firearm laws and interstate firearm deaths from homicide and suicide in the United States: a cross-sectional analysis of data by county. JAMA Intern Med. 2018;178(5):692-700. doi:10.1001/jamainternmed.2018.0190 29507953 PMC5885268

[25] Stone DM, Simon TR, Fowler KA, Vital Signs: trends in state suicide rates—United States, 1999-2016 and circumstances contributing to suicide—27 States, 2015. MMWR Morb Mortal Wkly Rep. 2018;67(22):617-624. doi:10.15585/mmwr.mm6722a1 29879094 PMC5991813

[26] Grant BF, Chou SP, Saha TD, Prevalence of 12-month alcohol use, high-risk drinking, and DSM-IV alcohol use disorder in the United States, 2001-2002 to 2012-2013: results from the National Epidemiologic Survey on Alcohol and Related Conditions. JAMA Psychiatry. 2017;74(9):911-923. doi:10.1001/jamapsychiatry.2017.2161 28793133 PMC5710229

[27] Han BH, Moore AA, Sherman S, Keyes KM, Palamar JJ Demographic trends of binge alcohol use and alcohol use disorders among older adults in the United States, 2005-2014. Drug Alcohol Depend. 2017;170:198-207. doi:10.1016/j.drugalcdep.2016.11.003 27979428 PMC5241162

[28] Masters RK, Tilstra AM, Simon DH Explaining recent mortality trends among younger and middle-aged White Americans. Int J Epidemiol. 2018;47(1):81-88. doi:10.1093/ije/dyx127 29040539 PMC6658718

[29] Diez Roux AV Despair as a cause of death: more complex than it first appears. Am J Public Health. 2017;107(10):1566-1567. doi:10.2105/AJPH.2017.304041 28902552 PMC5607706

[30] Muennig PA, Reynolds M, Fink DS, Zafari Z, Geronimus AT America’s declining well-being, health, and life expectancy: not just a White problem. Am J Public Health. 2018;108(12):1626-1631. doi:10.2105/AJPH.2018.304585 30252522 PMC6221922

[31] Dow WH, Godoy A, Lowenstein CA, Reich M Can economic policies reduce deaths of despair? Institute for Research and Labor Employment; 2019. Working paper No. 104-19. Accessed August 14, 2020. http://irle.berkeley.edu/files/2019/04/Can-Economic-Policies-Reduce-Deaths-of-Despair.pdf

[32] Gertner AK, Rotter JS, Shafer PR Association between state minimum wages and suicide rates in the U.S. Am J Prev Med. 2019;56(5):648-654. doi:10.1016/j.amepre.2018.12.008 30905484

[33] Ruhm CJ Deaths of despair or drug problems? National Bureau of Economic Research. Working paper No. 24188. Accessed August 14, 2019. https://EconPapers.repec.org/RePEc:nbr:nberwo:24188

[34] Knapp EA, Bilal U, Dean LT, Lazo M, Celentano DD Economic insecurity and deaths of despair in US counties. Am J Epidemiol. 2019;188(12):2131-2139. doi:10.1093/aje/kwz103 31172197 PMC7212405

[35] Sharfstein JM A New Year’s wish on opioids. JAMA. 2018;319(6):537-538. doi:10.1001/jama.2018.0058 29365013

[36] Dowell D, Haegerich TM, Chou R CDC guideline for prescribing opioids for chronic pain—United States, 2016. JAMA. 2016;315(15):1624-1645. doi:10.1001/jama.2016.1464 26977696 PMC6390846

[37] Stone D, Holland K, Bartholow B, Crosby A, Davis S, Wilkins N; National Center for Injury Prevention and Control Preventing suicide: a technical package of policy, programs and practices. Accessed August 14, 2020. https://www.cdc.gov/violenceprevention/pdf/suicideTechnicalPackage.pdf

[38] American Public Health Association Reducing suicides by firearms. Accessed September 25, 2019. https://www.apha.org/policies-and-advocacy/public-health-policy-statements/policy-database/2019/01/28/reducing-suicides-by-firearms

[39] Curry SJ, Krist AH, Owens DK, ; US Preventive Services Task Force Screening and behavioral counseling interventions to reduce unhealthy alcohol use in adolescents and adults: US Preventive Services Task Force recommendation statement. JAMA. 2018;320(18):1899-1909. doi:10.1001/jama.2018.16789 30422199

[40] Arias E, Heron M, Hakes J; National Center for Health Statistics; US Census Bureau The validity of race and Hispanic-origin reporting on death certificates in the United States: an update. Vital Health Stat 2. 2016;(172):1-21. 28436642

