# Supplementary material for: Trends in Mortality From Drug Poisonings, Suicide, and Alcohol-Induced Deaths in the United States From 2000 to 2017
Source: JAMA Netw Open. 2020 Sep 11;3(9):e2016217. doi: 10.1001/jamanetworkopen.2020.16217 (PMC7489841; doi:10.1001/jamanetworkopen.2020.16217)
Supplement: Supplement. — eTable 1. International Statistical Classification of Diseases and Related Health Problems, Tenth Revision (ICD-10) Codes for Drug Poisoning, Suicide and Alcohol-Induced Deaths eTable 2. P Values for Test of Parallelism eFigure. Trends in Drug Poisoning, Suicide and Alcohol-Induced Death Rates Among Individuals Aged 20 to 64 Years in the United States From 2000 to 2017 Stratified by Sex and Race/Ethnicity [file jamanetwopen-e2016217-s001.pdf]

Supplementary Online Content

Shiels MS, Tatalovich Z, Chen Y, et al. Trends in mortality from drug poisonings, suicide, and alcohol-induced deaths in the United States from 2000 to 2017. *JAMA Netw Open*. 2020;3(9):e2016217. doi:10.1001/jamanetworkopen.2020.16217

eTable 1. *International Statistical Classification of Diseases and Related Health Problems, Tenth Revision* (ICD-10) Codes for Drug Poisoning, Suicide and Alcohol-Induced Deaths

eTable 2. *P* Values for Test of Parallelism

eFigure. Trends in Drug Poisoning, Suicide and Alcohol-Induced Death Rates Among Individuals Aged 20 to 64 Years in the United States From 2000 to 2017 Stratified by Sex and Race/Ethnicity

This supplementary material has been provided by the authors to give readers additional information about their work.

© 2020 Shiels MS et al. *JAMA Network Open.*

eTable 1. *International Statistical Classification of Diseases and Related Health Problems, Tenth Revision* (ICD-10) Codes for Drug Poisoning, Suicide and Alcohol-Induced Deaths

Cause-of-death	ICD-10 Codes	Notes
Drug poisonings	X40-X44, Y10-Y14	Includes accidents and deaths with unknown intent
Suicides	U03, X60-84, Y87.0	Includes intentional poisonings due to drugs and alcohol
Alcohol-induced	E24.4, F10, G31.2, G62.1, G72.1, I42.6, K29.2, K70, K85.2, K86.0, R78.0, X45, Y15	Excludes intentional poisonings due to alcohol

© 2020 Shiels MS et al. *JAMA Network Open.*

eTable 2. *P* Values for Test of Parallelism

	**P-values for test of Parallelism**		
	Drug poisoning vs. suicide	Alcohol vs. drug poisoning	Suicide vs. alcohol
Overall	<0.001	0.004	<0.001
Men	<0.001	0.003	0.001
Women	<0.001	<0.001	<0.001
Ages 20-34	<0.001	0.02	<0.001
Ages 35-49	0.002	0.005	<0.001
Ages 50-64	<0.001	<0.001	0.001
White	<0.001	0.001	<0.001
Black	<0.001	<0.001	<0.001
Latino	0.001	0.002	0.03
Asian	<0.001	<0.001	0.22
American Indian/Alaska Native	<0.001	0.002	0.01

© 2020 Shiels MS et al. *JAMA Network Open.*

eFigure. Trends in Drug Poisoning, Suicide and Alcohol-Induced Death Rates Among Individuals Aged 20 to 64 Years in the United States From 2000 to 2017 Stratified by Sex and Race/Ethnicity

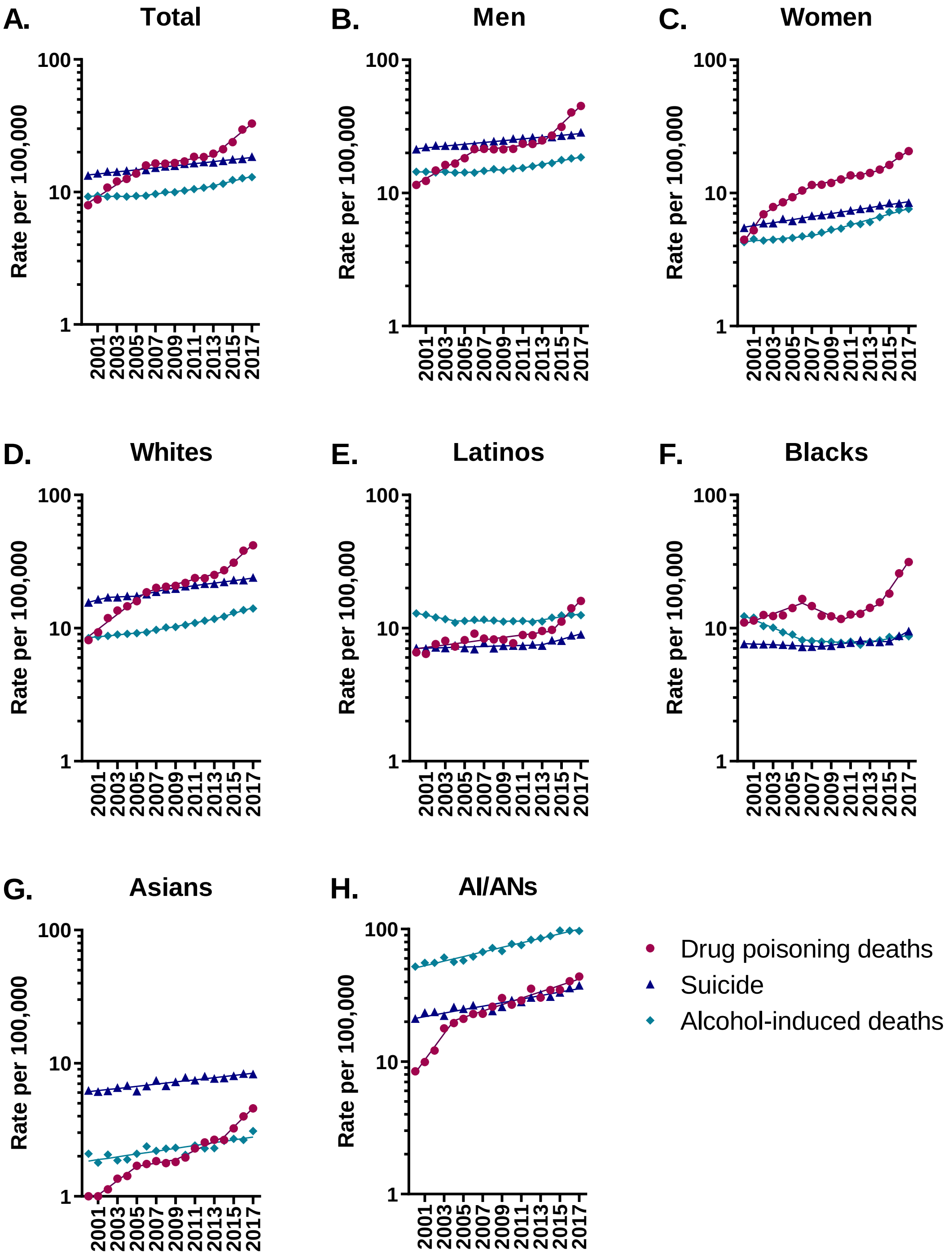

Points represent annual age-standardized death rates and lines represent modeled trends from Joinpoint models. The y-axis is a log10 scale.

© 2020 Shiels MS et al. *JAMA Network Open.*
